# The Computer-based Health Evaluation Software (CHES): a software for electronic patient-reported outcome monitoring

**DOI:** 10.1186/1472-6947-12-126

**Published:** 2012-11-09

**Authors:** Bernhard Holzner, Johannes M Giesinger, Jakob Pinggera, Stefan Zugal, Felix Schöpf, Anne S Oberguggenberger, Eva M Gamper, August Zabernigg, Barbara Weber, Gerhard Rumpold

**Affiliations:** 1 Department of Psychiatry and Psychotherapy, Innsbruck Medical University, Anichstr. 35, Innsbruck A-6020, Austria; 2 Evaluation Software Development OG, Feldstraße 2, Rum, A-6063, Austria; 3 Institute of Computer Science, University of Innsbruck, Technikerstraße 21a, Innsbruck, A-6020, Austria; 4 Oncotyrol – Center for Personalized Cancer Medicine GmbH, Karl-Kapferer-Straße 5, Innsbruck, A-6020, Austria; 5 Department of Internal Medicine, Kufstein County Hospital, Endach 27, Kufstein, A-6330, Austria; 6 Department of Medical Psychology, Innsbruck Medical University, Schöpfstraße 23a, Innsbruck, A-6020, Austria

## Abstract

**Background:**

Patient-reported Outcomes (PROs) capturing e.g., quality of life, fatigue, depression, medication side-effects or disease symptoms, have become important outcome parameters in medical research and daily clinical practice. Electronic PRO data capture (ePRO) with software packages to administer questionnaires, storing data, and presenting results has facilitated PRO assessment in hospital settings. Compared to conventional paper-pencil versions of PRO instruments, ePRO is more economical with regard to staff resources and time, and allows immediate presentation of results to the medical staff.

The objective of our project was to develop software (CHES – Computer-based Health Evaluation System) for ePRO in hospital settings and at home with a special focus on the presentation of individual patient’s results.

**Methods:**

Following the Extreme Programming development approach architecture was not fixed up-front, but was done in close, continuous collaboration with software end users (medical staff, researchers and patients) to meet their specific demands. Developed features include sophisticated, longitudinal charts linking patients’ PRO data to clinical characteristics and to PRO scores from reference populations, a web-interface for questionnaire administration, and a tool for convenient creating and editing of questionnaires.

**Results:**

By 2012 CHES has been implemented at various institutions in Austria, Germany, Switzerland, and the UK and about 5000 patients participated in ePRO (with around 15000 assessments in total). Data entry is done by the patients themselves via tablet PCs with a study nurse or an intern approaching patients and supervising questionnaire completion.

**Discussion:**

During the last decade several software packages for ePRO have emerged for different purposes. Whereas commercial products are available primarily for ePRO in clinical trials, academic projects have focused on data collection and presentation in daily clinical practice and on extending cancer registries with PRO data. CHES includes several features facilitating the use of PRO data for individualized medical decision making. With its web-interface it allows ePRO also when patients are home. Thus, it provides complete monitoring of patients‘physical and psychosocial symptom burden.

## Background

Since the 1990s there has been growing awareness of the importance to complement the traditional physician-rated assessment of the patients’ health status by an assessment made by the patients themselves, so-called patient-reported outcomes (PROs) [1-4]. These PROs include physical and psychosocial symptoms (e.g., side-effects, quality of life (QOL), gastrointestinal symptoms, fatigue, pain, anxiety, and depression).

By now advanced computer technology widely available at medical institutions allows a reduction of required human resources and makes the routine collection of data feasible in busy clinical practices [5-8]. Also, computer technology is a necessary requirement for immediate and sophisticated presentation of PRO results to clinicians.

An important issue related to electronic administration of PRO instruments is the equivalence of scores derived from electronic and from paper-pencil questionnaires. A meta-analysis by Gwaltney et al. [9] suggests that psychometric properties are rather invariant across modes of administration and scores do not differ substantially. This is in line with a report of the ISPOR ePRO Good Research Practices Task Force report that states, that minor modifications of questionnaire design are not likely to introduce response bias or to impact psychometric properties [10]. A comprehensive theoretical overview on factors potentially affecting comparability of scores and questioning score equivalence is given by Robling et al. [11].

Within various medical fields, especially within oncology, there are several research groups that engaged in programming and implementing software solutions for electronic PRO data capture (ePRO) in clinical routine in recent years [12-14]. Also, web-based PRO assessments extending data capture beyond the hospital setting have been implemented by a few research groups [15-17]. Recently, ISOQOL published guidelines on PRO assessments in clinical routine [18]. These guidelines, which are expected to be updated periodically, provide a helpful overview on (dis)advantages and required resources related to various implementation options.

Reviews by Luckett et al. [13] or Greenhalgh [19] provides an overview of studies evaluating the benefit of such software packages for symptom monitoring and health outcome in oncological patients. But also in other medical fields studies investigating or using electronic PRO data capture software are available from the literature. For example, Crane et al. [20] showed feasibility of touch-screen based PRO data collection in HIV patients. Kinnaman et al. [21] developed the Computerized Assessment System for Psychotherapy Evaluation and Research (CASPER) for use in psychiatric patients. Earlier, Stern et al. [22] evaluated the computer-driven patient self-rated questionnaire (COSAPSQ) for the same patient group.

These software tools using touch screens for questionnaire administration have shown feasibility in daily clinical practice and implementation studies suggest important benefits for the physicians, the patients and medical care [12,23,24]. A detailed model on how provision of PRO data may influence medical decision making has been introduced by Greenhalgh et al. [25]. Potential benefits include, e.g., screening for potential health and/or psychosocial problems, facilitating communication and shared clinical decision making, monitoring changes or response to treatment, enhancing patients’ quality of life (QOL) and satisfaction with care, and changing and improving patient management and treatment outcome [25]. Some of these advantages are quite evident and proved by the above mentioned, well-designed randomized controlled trials, while others have to be investigated further [19].

In this article, we want to present the software CHES - Computer-based Health Evaluation System. CHES is a software for electronic PRO questionnaire administration, storage of PRO and clinical/sociodemographic data and for the graphical presentation of PRO results in relation to medical data. It comprises important features like the administration of computer-adaptive PRO tools, web-based questionnaire completion, and data import/export. This software has been developed by the academic spin-off company Evaluation Software Development (ESD). Recently, the software development efforts have been intensified through a joint project with the EORTC Quality of Life Group that also partly funds further software development through a research grant. The collaboration aims at making a software tool available for QOL studies and for daily clinical practice.

In this paper, we will describe and discuss the following issues related to our software CHES: user group considerations, software development, key features of CHES and implementation into daily clinical routine.

### Users, software development and features of CHES

#### User group considerations

**Figure 1:**
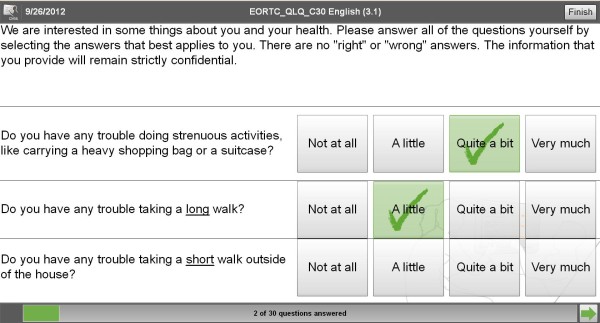
Questionnaire (EORTC QLQ-C30) as presented to patients in CHES.

**Figure 2:**
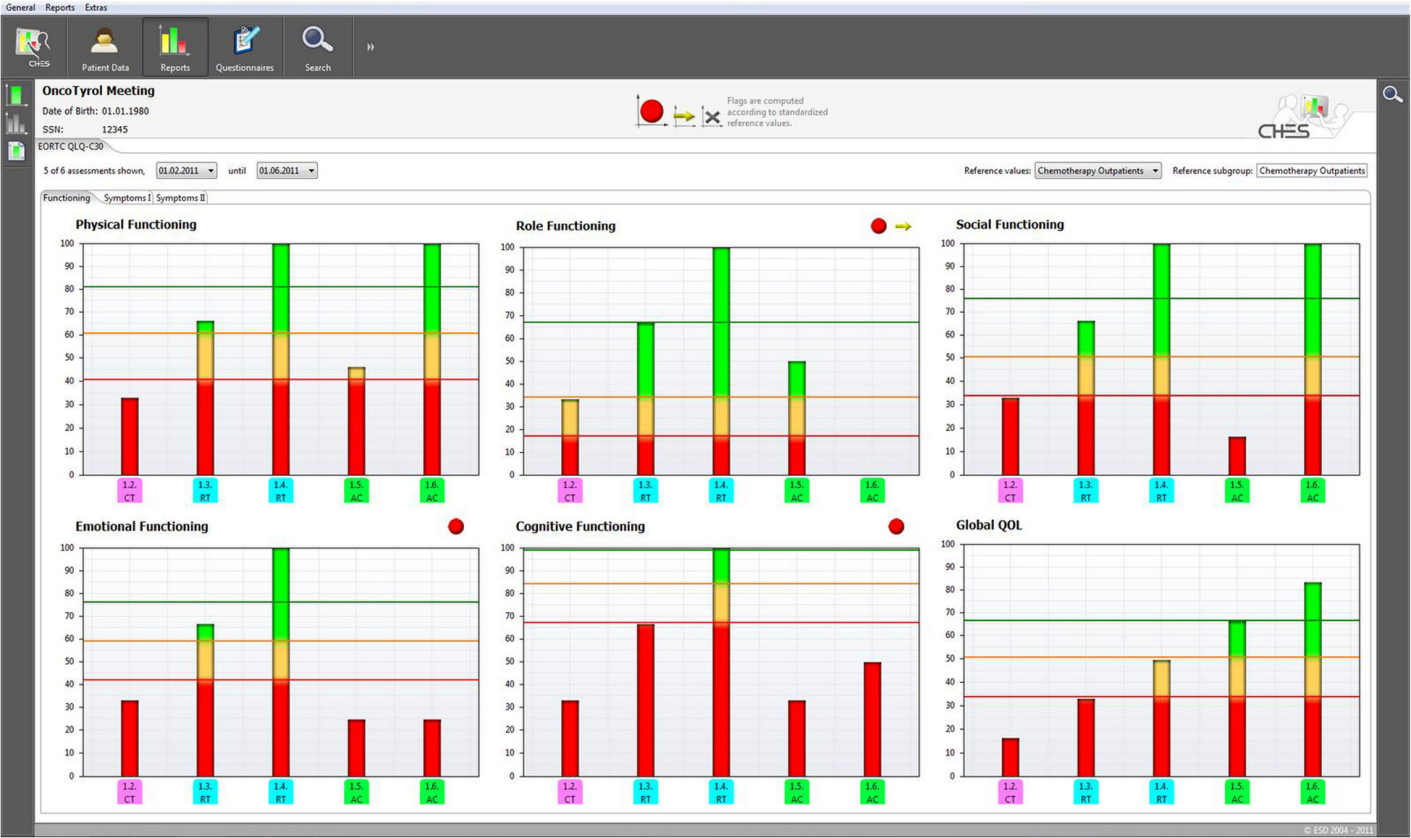
Clinical PRO report in CHES.

As pointed out in the introduction, CHES was designed to foster the integration of PROs in daily clinical routine. To develop an appropriate IT solution for this scenario, it is of utmost importance to constantly take the application's end users’ feedback into account [26]. Usability prototyping and usability testing ensure that users are included at an early stage (standards for user oriented software are specified in ISO 13407). It is crucial to tailor the software to the specific needs and abilities of potential users. We identified several user groups for CHES leading to specific requirements on the availability of certain features for each of them:

• The patient completes questionnaires on the tablet-PC requiring easy handling of the data entry interface (see Figure 1). The patient's motivation is to provide the physician with PRO data enabling him/her to better account patient's subjective health status within medical decision making. For questionnaire completion patients are only required to read the text on the screen (font size can be adapted) and select the appropriate response categories, i.e. in case of tablet PCs to put their finger on the response category. As questionnaire pages turn only if a patient answered all questions on the page (otherwise they have to be turned by pressing a button), patients cannot omit questions unintentionally. This decreases number of missing items in questionnaire results.

• The physician needs information on the patient's current health status and its development over the disease trajectory. This information should be easily interpretable and should be linked to medical data and clinically relevant events (see Figure 2).

• The researcher is interested in high-quality PRO data, in particular in longitudinal data with a low proportion of missing values. Longitudinal data collection is facilitated by integrating PRO data collection into clinical routine. IT support is necessary for standardized data collection. PRO data and medical data should be stored in a central database. The software should provide means for exporting data to common statistical software packages. In addition, the software should provide study monitoring with easy inclusion of clinical report forms and a quick overview on missing data.

• The nurse, study nurse or intern approach the patient and provide help with questionnaire completion. They need support for the administration of PRO questionnaires and they are disburdened from data entry, scoring and generating of reports.

• The chief physician is interested in implementing ePRO in clinical routine practice to improve medical decision making, to foster research activities and to optimize quality assurance.

• The system administrator is responsible for setting up, configuring and updating the software. S/he is interested in a stable software solution going along with a long-term perspective regarding support and further development. Furthermore, s/he needs a tool for editing and importing PRO-questionnaires into the software.

As pointed out these user groups differ in what features of the software they use. As a consequence, the various features should be tailored to the specific user group requirements. This means that for example the patient front-end of the software should be as simple as possible, while the administrator area may be rather complex and provide comprehensive configuration details. The physician front-end should be designed in an easy to use, but still visually appealing way, allowing quick access to all relevant patient data. In this context, an important issue is providing high usability to computer illiterate people.

#### Development process

For this project Extreme Programming (XP) [26] has been adopted as software development approach. XP is considered a flexible approach to planning, making it well suited to react on changed requirements during the course of the project. As suggested in XP, the CHES development team adopted an incremental software architecture to match the demands of the various user groups. More specifically, the software architecture was not fixed upfront, but rather evolved over time. For this purpose, periodic releases and resulting feedback allowed for driving the development of CHES in a direction, which ensured the acceptance in daily clinical routine.

Software quality control was done by utilizing automated unit test for automatically testing the software’s features and by using an issue tracking system for keeping track of reported problems. The issues tracking system was also used for driving the development process by recording feature requests which were subsequently discussed and documented in face to face meetings between physicians and developers. In addition, a build server, i.e., a dedicated server responsible for incorporating the latest changes and building the software, ensures the constant availability of the latest version to developers and testers.

After developing a proof of concept version of CHES in 2005 the development team switched to a two-tier approach—a client application accessing a central database. At the same time, the used programming language switched from Visual Basic to Java based on the Eclipse Rich Client Platform (RCP). In 2009 the graphical user interface of CHES was substantially revised to optimize usability and attractiveness of the software.

#### Key features of CHES

This section sketches major features of CHES ranging from the creation of questionnaires for data capture to the graphical presentation of results to physicians and export of collected data for scientific studies.

### Creating and editing questionnaires

• Questionnaire Builder: CHES Questionnaire Builder was developed for defining the structural properties (i.e., question and answer texts, or psychometric item characteristics) of questionnaires. In addition, CHES Questionnaire Builder enables researchers to define the visual appearance of questionnaires in order to adapt them to different devices, e.g., tablets, smart phones, or specific patient groups (e.g., elderly people, visually impaired).

### Electronic data capture

• Survey Application: Within daily clinical practice or clinical studies, the survey application presents questionnaires to patients, preferably using a touch screen device, e.g., tablet. Special care has been taken to provide a simple and intuitive, yet configurable user interface in order to ease patient-computer interaction. Figure 1 shows a screenshot of the questionnaire as it is displayed to the patient.

• Home Monitoring: Web-based home monitoring extends ePRO beyond clinical settings, thus completing information on the patient's health status. This is not only important to track symptom burden over the treatment or disease trajectory, but even more so to be able to provide interventions when needed. Web-based assessments also facilitates the management of data collection in international multi-centre trials considerably.

• iPad Application CHES. Survey: To administer questionnaires also on iPads’ we have developed an application in close collaboration with the Austrian software company WorldDirect.

• Online case report forms: To facilitate multi-centre studies CHES not only provides online presentation of questionnaires but also a researcher front-end allowing managing patients in a study and completing case report forms online.

• Computer-adaptive testing (CAT): The development of computer-adaptive testing (CAT) of various PRO measures is currently a major research focus in this field of PRO research [27,28]. CAT uses item response theory models to generate tailored item sets for individual patients. Based on an initial item an algorithm selects the subsequent item from an itembank based on the individual’s previous response. The item is chosen in such a way that the information gained is maximized. CAT reduces test length considerably without loss of precision [29], i.e., assessment duration as well as patient burden is minimized. CHES has been extended to accommodate for this new type of PRO assessment and to seamlessly integrate it into daily clinical routine. Currently, we have implemented the Expected a Posteriori (EAP) estimation method for calculating scores. For CAT CHES allows to define the starting item, the minimum and maximum number of items to be asked, and the width of the 95% confidence interval of the score at which the CAT procedure stops (based on the Maximum Fisher Information Function). These settings can be changed in the CHES administrator area.

• Study Monitoring: The gathering of medical as well as PRO-data in the course of studies is supported. For this purpose, Study Monitoring provides means of highlighting data to be collected as well as tracking of missing study-related data.

### Data analysis and interpretation

• Graphical presentation of results: results are presented as colored graphs in real time. The graphical output (see Figure 2) links PRO to the course of disease and treatment and in addition specific medical interventions can be easily incorporated and displayed. Results can be displayed optionally in a longitudinal or cross-sectional setup.

• Flag System: Based on reference values from literature or previously collected data, the Flag System allows for the quick identification of patients with clinically relevant problems, using cut-off scores or score distributions.

• Clinical Report Generator: CHES supports the automatic generation of clinical reports, including questionnaire results represented using charts as well as clinical data. For interoperability, reports can be stored as Portable Document Files (PDF).

### Interfaces for data exchange

• Data Export/Import: Sociodemographic, clinical and questionnaire data can be exported to different file formats and imported from files, e.g., SPSS or MS-Excel.

• Interface to clinical information systems (HL7): In order to exchange medical and sociodemographic data between CHES and clinical information systems (CIS) a HL7-interface is available.

### General features

• Multilingualism: The whole life-cycle — designing questionnaires, administering questionnaires and displaying the results to physicians — was designed to accommodate for the need of supporting different languages. Therefore, nurses are able to select the language for questionnaire administration for each patient individually. The software is currently available in English, German and Italian. Further translations are currently ongoing.

• User Management: Depending on the task and requirements of the specific clinical or research setting different user rights are granted.

• Update Mechanism: In order to efficiently install updates for local CHES installations, an update mechanism is in place, providing an one-click solution for installing, new features and available bug fixes.

#### Data security

Confidentiality is one of the main design goals of CHES. However, as pointed out by Chang [30], the avoidance of staff burden represents an important factor for the acceptance of PRO. Consequently, a security concept has been devised which ensures the confidentiality of data without forcing its user to hurdle any unnecessary burdens and leaving an acceptable residual risk.

**Figure 3:**
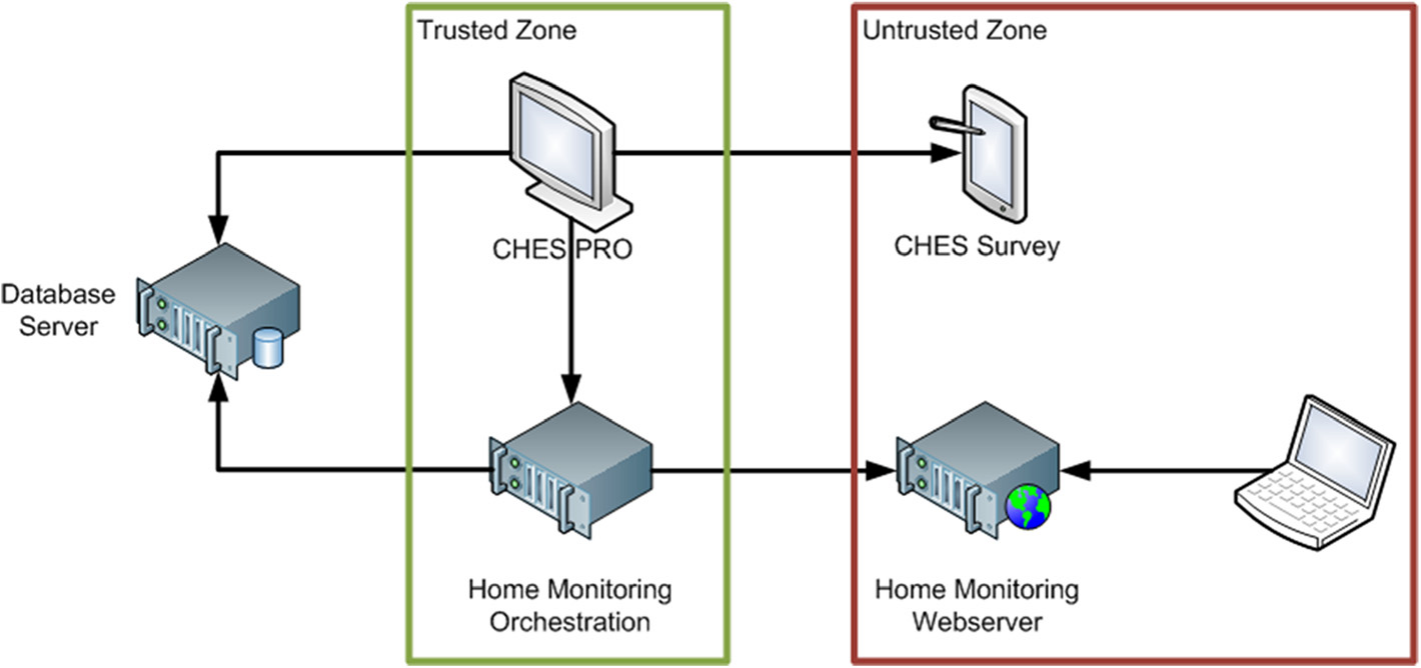
CHES Architecture.

The security concept builds upon existing security infrastructure provided by the operating system forming a so-called trusted zone that only authorized users may have access to, e.g., CHES PRO running on a physician’s computer, restricted by user accounts of the operating system (see Figure 3). Untrusted zones on the other hand can be accessed by anyone, containing webservers running the home monitoring web application and tablets running CHES Survey, which are available to patients in waiting rooms of hospitals. This separation led to the following three design decisions.

• Divide the CHES Toolset in applications which run in trusted and untrusted zones.

• Minimize access to data in untrusted zones.

• Provide a secure mean of communication between trusted and untrusted zones.

Within the trusted zone CHES PRO can access restricted resources, e.g., the database. CHES Survey on the other hand, cannot connect to the database as it is in the untrusted zone and can only communicate with CHES PRO via an XML interface, e.g., through the exchange of files. The XML file is encrypted using a secure Advanced Encryption Standard (AES) cipher [31] to provide a secure mean of communication. This ensures that a minimum amount of data is securely transferred to the untrusted zone.

In a similar vein, the web server hosting the home monitoring web application cannot access the database server as it is situated in the untrusted zone. For this purpose, the home monitoring orchestration server is responsible for pushing questionnaires to be administered to the webserver on a regular basis, e.g., as described in the study the patient is participating in, or on an ad-hoc basis triggered by a nurse or physician using CHES PRO. Again, only a minimal amount of data is transferred to the untrusted zone to guarantee a maximum amount of data security.

#### Implementation

By 2012 CHES has been implemented in a number of hospital settings in various fields of medicine (e.g., urology, nephrology, orthopedics, radiation therapy, neurology, oncology, gynecology, health psychology) in Austria, Germany, Switzerland, and the UK. So far, about 5000 patients completed the computerized questionnaires with a total of approximately 15000 assessments. Its web-interface for completing questionnaires and case report forms online is currently used in an international EORTC questionnaire validation study (EORTC QLQ-TC26) in five European countries and Australia, and in a CAT validation study in Denmark.

At these centers data assessment is done via tablet-PCs with screen sizes of 10" or 12". Usually one to four questions depending on screen size and patient’s eyesight are shown at once. In most settings, a client–server solution for data storage is in place based on either Wi-Fi or LAN. This is necessary in order to provide results without time loss at all working stations required.

The selection of implemented PRO questionnaires depends on patients’ disease/treatment, study objectives and availability of copyrights. Given that currently CHES is mostly used at units treating oncological patients, we rely strongly on the QOL questionnaires developed by the EORTC Quality of Life Group [32]. The internationally widely used EORTC questionnaires allow the comprehensive and valid assessment of functioning and symptoms in cancer patients by combining a generic questionnaire with disease-specific modules.

To provide specific experiences with implementation of CHES in clinical settings, we describe the successful implementation at two hospitals in Austria.

##### Inpatient unit of the Department of Internal Medicine at Kufstein County Hospital

ePRO has been ongoing since 2004 and ~4500 assessments have been conducted so far. The patients treated at this unit are diagnosed with various cancer types and are receiving chemotherapy.

Data collection is performed by a study nurse who also supervises questionnaire completion. Supervision includes explanation on specific questions, as well as explanations on how to fill in the questionnaire on the tablet PC. In visually impaired patients or patients with motor dysfunctions the study nurse conducts the questionnaire as an interview and enters the patient’s responses. According to the time frame of the employed questionnaires, the time interval between two assessments is at least one week.

As Wi-Fi is currently not available at this unit, CHES is connected via LAN to the database. This means, that the study nurse connects the tablet-PC to the LAN and prepares the questionnaire to be administered. Then the study nurse disconnects the tablet-PC for bed-side assessments. After data collection the study nurse connects again with the LAN and uploads the data to the database. As patient-physician contacts in this inpatient setting are not as limited as in an outpatient unit, the immediate availability of results is not considered crucial. The results from the EORTC QLQ-C30 are mainly used to screen for potential clinically relevant problems (e.g. treatment side-effects and symptom burden). The treating physician uses the graphical PRO result presentation within patient-physician contact to start discussion about specific issues or to talk about changes in health condition. Physicians and nursing staff involved in ePRO have been trained in an one hour training session to interpret the results from the QLQ-C30 which is facilitated by linking the results to reference scores. This session is repeated periodically and includes detailed discussion of specific QOL profiles from individual patients seen at that unit.

Since June 2012 we have extended data collection to the time between hospital visits using the above mentioned CHES iPad Application. Patients willing to participate in this kind of PRO home monitoring currently receive iPads from the hospital for the duration of chemotherapy.

##### Outpatient unit of the Department of Urology at Innsbruck Medical University

ePRO has started in 2007 and 800 assessments have been done using CHES since then. Testicular cancer patients in aftercare after chemotherapy and/or surgery are included in ePRO. Testicular cancer patients are a relatively young patient group with survival rates of almost 100% [33].

Medical staff was trained in handling the software and interpretation of results right from the beginning. Assessments take place in a secluded area of the waiting room while patients are waiting for their aftercare appointment with the physician. A psychological intern approaches the patient and explains the purpose of ePRO as well as further issues related to confidentiality and questionnaire completion. If willing to participate, a tablet-PC is handed to the patient showing the questionnaires on its screen. While filling questionnaires by tipping on the response categories, the intern is available for any questions arising.

After completing the questionnaire in CHES the intern collects the tablet-PC and transfers the data via Wi-Fi to the database. Thus, the PRO data is immediately available to the treating physician when the patient enters the room. Similar to Kufstein County Hospital PRO data (EORTC QLQ-C30 and QLQ-TC26) is used for symptom screening and monitoring and within patient-physician communication.

Data collected within the routine ePRO is also used for research purposes. In addition, the ePRO logistics are used aside of clinical routine for ongoing clinical studies.

So far two CHES implementations into daily clinical routine failed. At both departments (treating oncological and transplantation patients at Innsbruck Medical University) we started with using CHES for clinical studies. However, after successful data collection for study purposes we did not manage to integrate the use of the PRO results into individual patient treatment. In personal communication, treating physicians reported the lack of additional value gained from PRO scores, which consequently did not justify the efforts for data collection.

### Related work

Currently, a number of projects developing and providing software solutions for ePRO are ongoing. These software packages differ relevantly with regard to the intended use of the collected PRO data. Commercial products are available for ePRO within clinical trials [34-36] allowing data collection via desktop computers, tablet-PCs, smart phones or interactive voice response (IVR) systems. A sophisticated platform allowing PRO data collection via the web-browser is offered by the US American initiative PROMIS, which is developing Item Response Theory-based PRO measures (including computer-adaptive instruments) [37]. This platform is free of charge and requires only registration of the institution intending to use it. However, graphical presentation of individual patients’ PRO results is rather rudimental.

ePRO at a wider, potentially nationwide level is pursued by two projects in the UK and the Netherlands. In the UK, the ePOCS system (electronic Patient-reported Outcomes from Cancer Survivors) has been established aiming not only at ePRO but also on the linkage of these data to existing cancer registries, making patient recruitment and tracking a major issue [38,39]. The Dutch project PROFILES (Patient Reported Outcomes Following Initial treatment and Long term Evaluation of Survivorship registry) has a similar purpose emphasizing psychosocial aspects of cancer survivorship and including also a large general population sample to gain reference scores for interpretation of the results from cancer survivors [40]. PROFILES include screening for individual patients potentially requiring medical or psychosocial interventions due to their mental or physical status. Thus, it combines large scale data collection for cancer registries with symptom management in the individual patient.

Another project from the Netherlands developed two software packages called OncoCompass and Oncoquest [41,42] that provide ePRO and individual PRO results presentation embedded. Whereas Oncoquest does not provide web-based data collection, Oncocompass is a comprehensive patient web-portal for research purposes which comprises further features such as personal health record, stepped care programs, and specific information on disease and treatment.

A comparable web-platform called STAR (Symptom Tracking and Reporting) is in use at the Memorial Sloan-Kettering Cancer Center to track symptom burden in patients after radical prostatectomy [16]. Another example in the field of pediatric oncology is the QLIC-ON software which has been successfully implemented and positively evaluated in a multi-centre study in the Netherlands [43] and includes a web-interface for data collection [44].

In comparison to these projects, CHES has a stronger focus on graphical presentation of individual PRO results in relation to medical data. It presents longitudinal and cross-sectional PRO charts, in which the PRO scores are automatically color-coded based on reference scores from specific patient groups (patients with e.g., same age, sex, diagnosis, and treatment phase). This facilitates considerably the results interpretation by medical staff not being familiar with this kind of data. Based on these reference scores, CHES also provides an alert system notifying the medical staff in case of clinically relevant impairments with pop-up messages. If activated, pop-up messages appear on the doctors’ screen as soon as a patient has entered PRO scores exceeding predefined limits. These messages include patient name, ID and birth date and a link allowing easy access to PRO results of this specific patient. The intended use is that the treating physician receives this information as soon as a patient has completed the questionnaire (e.g. while waiting for his/her appointment). In a busy clinical setting, it may be beneficial to be informed only about patients with clinically relevant problems and not having to look at every single patient chart.

## Discussion

Electronic PRO data capture (ePRO) in daily oncological practice or clinical trials allows to measure subjective physical health status and psychosocial burden. Within clinical routine it provides additional information for medical decision making, helps to monitor symptom burden and improves patient-physician communication. In a research context, PROs have become an important outcome supplemental to clinical parameters (e.g., survival, toxicity ratings). Advantages of ePRO are the reduced need of human resources for data collection making it economically efficient and the increased data quality as paper-based systems account for a certain proportion of variance in medical outcomes [45].

Early calls for software tools in order to improve accessibility and management of medical information [46] were followed by the development of systems to facilitate not only collecting data but also synthesizing and analyzing outcomes. With the increasing use of PRO data the need for adequate software packages became more urgent. In principle, this need can be met by programming extensions for existing clinical information systems or by creating a new software package. An extension provides the advantage of a native solution not requiring interfaces for data exchange. Naturally, plug-ins for existing clinical information systems are rather environment-specific and require comprehensive adaptation when implementing into another IT environment. In contrast, programming a new software package, which was our approach, allows a high degree of flexibility for feature development and easier software dissemination.

When developing CHES we focused especially on issues like ease of use, the graphical presentation of individual PRO scores, and data security. We considered ease of use as crucial as it is known from literature, that perceived ease of use affects the perceived usefulness and, consequently, the usage of information technologies [47]. Therefore, we believe that ease of use is likely to have an impact on acceptance by the medical staff and the hospital management. The graphical presentation of individual PRO is of little relevance in a research context where the focus is primarily on mere data collection and statistical analysis. However, in daily clinical practice PRO scores from the individual patient are of importance and since medical staff is frequently not very familiar with the interpretation of individual PRO scores, the need for software facilitating this task is obvious.

A limitation of the software CHES is that the current Survey Application relies on Adobe Flash runtimes to display questionnaires. This limits its use as Adobe Flash runtimes is not supported by iOS and may become less common in the near future. The above mentioned iPad Application helps to partly overcome this limitation. Furthermore we are currently reprogramming parts of our software to use HTML5 for questionnaire display, to be less platform dependent. Also, CHES currently provides only bar charts for presenting PRO results, which may not meet all user preferences and also makes it difficult to show several PRO scores in one chart. The latter may be important for synopsis of several PRO scores.

In general, implementation of ePRO systems are confronted with some problems and barriers mostly related to resources (time and money). These are related to e.g. purchasing or developing an ePRO system, adapting the software to requirements of a specific hospital setting, providing the hardware needed for data collection, software and database maintenance, and for staff required for supervising questionnaire administration. Whereas patient acceptance is usually high [48-50], physicians’ attitude towards and knowledge about PRO data and their potential benefits is sometimes skeptical [51]. As mentioned above we consider the attitude of medical staff towards ePRO to be a key factor in integrating it into daily clinical practice.

Feasibility itself is reported to be less a problem [24,52,53]. With regard to CHES, in a recent study on the feasibility of PRO assessments in 60 patients after total hip or knee arthroplasty, 98% of the patients reported no difficulties with completing electronic questionnaires presented within CHES on a tablet PC, although about half of the patients indicated to never use a computer in daily life [54]. In an earlier study on the implementation of CHES at the neurooncological outpatient unit [8] at Innsbruck Medical University we found that only 3.6% of the patients were not willing to complete questionnaires electronically, whereas in that specific patient group 20.0% were not capable of participation due to brain tumor-related impairments. Also, that study showed that time for questionnaire completion dropped by 30% from the first to the fifth assessment.

Further development of our software will include its adaptation to allow questionnaire administration on smart phone web-browsers (using HTML5). Smart phones are attractive data collection tools, as some patients may have easier access to their smart phone than to a PC. Adaptation of the questionnaires to smart phones will primarily focus on graphical features of questionnaire presentation, e.g. due to screen size only one item will be shown at once.

Another focus will be the programming of interfaces to clinical information systems allowing to export clinical PRO reports generated in CHES into these systems. This is a challenging issue, as clinical information systems differ considerably between hospitals requiring customized data exchange interfaces.

## Conclusion

In conclusion, the software CHES provides a useful tool for PRO data collection and presentation. It is applicable for research purposes, for symptom screening and monitoring in the individual patient, and contributes to quality assurance in hospitals.

## Availability and requirements

• Project name: Computer-based Health Evaluation System (CHES)

• Project home page: <http://www.ches.at>

Operating system(s): Windows XP, Vista, 7

• Programming language: Java

• Other requirements: Java 6*, Adobe AIR Runtime*

• License: Free version for non-commercial use; full functionality; not supporting Client–server Settings; proprietary license for Client–server Setting

• Any restrictions to use by non-academics: Proprietary License bundled with CHES, no separate installation required

• Download link: http://ches.at/ches/release/eortc/standalone/CHES_Standalone_25022011

• Demo for online questionnaire administration: http://homemonitoring.ches.at?demo=true user name: reviewer password: reviewer

## Competing Interests

Bernhard Holzner and Gerhard Rumpold are owner of the intellectual property rights of the software CHES.

## Authors' contributions

BH and GR conceived the original design of the software, programmed early software versions and supervise software development. JP, SZ and FS programmed the current version of the software. FS and GR provide software support, manage all technical problems and fix bugs in the software. AZ, JMG, ASO, EMG and BH were responsible for software testing and implementation into daily clinical practice and into research projects. All authors contributed to software development by conceiving software features. BH, GR, FS, JMG, SZ, JP, EMG and BW participated in writing of the manuscript. All authors read and approved the final manuscript.

## Pre-publication history

The pre-publication history for this paper can be accessed here:

http://www.biomedcentral.com/1472-6947/12/126/prepub
